# Single-Molecule Imaging Reveals the Activation Dynamics of Intracellular Protein Smad3 on Cell Membrane

**DOI:** 10.1038/srep33469

**Published:** 2016-09-19

**Authors:** Nan Li, Yong Yang, Kangmin He, Fayun Zhang, Libo Zhao, Wei Zhou, Jinghe Yuan, Wei Liang, Xiaohong Fang

**Affiliations:** 1 Beijing National Laboratory for Molecular Sciences, Key Laboratory of Molecular Nanostructure and Nanotechnology, Institute of Chemistry, Chinese Academy of Sciences, Beijing 100190, P.R. China; 2 University of Chinese Academy of Sciences, Beijing, 100049, P.R. China; 3 Key Laboratory of Protein and Peptide Drugs, Institute of Biophysics, Chinese Academy of Sciences, Beijing 100101, P.R. China

## Abstract

Smad3 is an intracellular protein that plays a key role in propagating transforming growth factor β (TGF-β) signals from cell membrane to nucleus. However whether the transient process of Smad3 activation occurs on cell membrane and how it is regulated remains elusive. Using advanced live-cell single-molecule fluorescence microscopy to image and track fluorescent protein-labeled Smad3, we observed and quantified, for the first time, the dynamics of individual Smad3 molecules docking to and activation on the cell membrane. It was found that Smad3 docked to cell membrane in both unstimulated and stimulated cells, but with different diffusion rates and dissociation kinetics. The change in its membrane docking dynamics can be used to study the activation of Smad3. Our results reveal that Smad3 binds with type I TGF-β receptor (TRI) even in unstimulated cells. Its activation is regulated by TRI phosphorylation but independent of receptor endocytosis. This study offers new information on TGF-β/Smad signaling, as well as a new approach to investigate the activation of intracellular signaling proteins for a better understanding of their functions in signal transduction.

TGF-β/Smad signaling pathway plays a pivotal role in a variety of important biological processes, including cell growth, differentiation, apoptosis, embryonic development and extracellular matrix formation^1^,^2^,^3^. Its dysregulation has been related to several human diseases such as cancer and tissue fibrosis^4^,^5^. The signaling is generally initiated with ligand-induced heteromerization of two membrane receptors, TGF-β type II receptor (TRII) and TGF-β type I receptor (TRI), and then transduced from cell membrane to nucleus by intracellular Smad proteins. Three classes of Smad proteins with different functions are involved: receptor-activated Smads (R-Smads, Smad2 and Smad3), common-mediator Smad (Co-Smad, Smad4) and inhibitory Smads (I-Smads, Smad6 and Smad7)^6^,^7^,^8^.

R-Smads are the key intracellular mediators to propagate TGF-β signal. According to the current knowledge on TGF-β signaling, R-Smads are predominantly localized in the cytoplasm of unstimulated cells and associated with the protein SARA (Smad Anchor for Receptor Activation). Upon TGF-β stimulation, SARA presents R-Smads to the phosphorylated TRI for activation. After phosphorylation of their C-terminal Ser-Ser-X-Ser (SSXS) motif by TRI, R-Smads dissociate from SARA with concomitant binding to Co-Smad, followed by nuclear translocation to regulate target gene expression^9^,^10^. Although the successive events in R-Smads activation and signaling have been established and the shuttling of R-Smads from cytoplasm to nucleus in response to TGF-β stimulating has been well studied^11^,^12^,^13^, the transient process of R-Smads activation by TRI is less understood. There are currently three different views on R-Smads activation: (i) The R-Smads bind to the activated TRI via SARA at plasma membrane, where the R-Smads are phosphorylated by TRI, then dissociated from the membrane for nuclear translocation^14^. (ii) After the binding of R-Smads with TRI at the cell membrane, the whole signaling complex including TGF-β receptors and R-Smads, is endocytosed into the early endosomes, followed by R-Smads phosphorylation, dissociation and nuclear translocation^15^,^16^. (iii) Upon ligand binding, the heteromeric TGF-β receptor complex is internalized into early endosomes, then the receptor-binding, phosphorylation and dissociation of R-Smads all take place in early endosomes instead of at cell membrane^17^. Besides the inconsistent results on the activation location (cell membrane or early endosomes in cytoplasm), the regulation mechanism of R-Smads activation remains unclear. The above inconsistent and incomplete knowledge on R-Smads activation is mainly due to the limitation of former *in vitro* biochemical assays, such as co-immunoprecipitation (Co-IP) using cell lysates or immunofluorescence with fixed cell, which measured averaged properties of ensemble R-Smads with low time and spatial resolution. As there are increasing evidences on the important role of R-Smads in disease progress including TGF-β-mediated induction of epithelial-mesenchymal-transition (EMT), breast cancer metastasis and fibrotic disorders^18^,^19^,^20^,^21^, understanding the transient and heterogeneous process of R-Smads activation is of critical importance. We thus designed a new approach to directly monitor the dynamics of Smad3 activation in living cells using single-molecule total internal reflection fluorescence microscopy (TIRFM).

Live-cell single-molecule TIRFM is an emerging technique to probe the structure and dynamics of signal transduction proteins, such as growth-factor receptors and ion channels^22^,^23^,^24^,^25^,^26^,^27^. Although this technique is mainly used to investigate membrane proteins, it is recently being explored to study the turnover of intracellular SH2 on cell membrane and membrane translocation of small GTPase Rac1^28^,^29^. In this work, we applied single-molecule TIRFM and robust single-molecule tracking method to image and quantify the dynamics of a typical R-Smad protein, Smad3. Membrane association and dissociation of enhanced green fluorescent protein (EGFP)-tagged single Smad3 molecules were monitored before and after TGF-β1 stimulation, and its change in docking dynamics is characterized for its activation study. We revealed, for the first time, the activation dynamics of Smad3 occurred on the plasma membrane via bindings with inactivated and activated TRI respectively, and verified it was endocytosis-independent. Our results provide new insights on Smad3 activation for a better understanding of this critical step in transducing TGF-β signal.

## Results

### Docking of Smad3 to cell membrane in both unstimulated and stimulated cells

**Figure 1:**
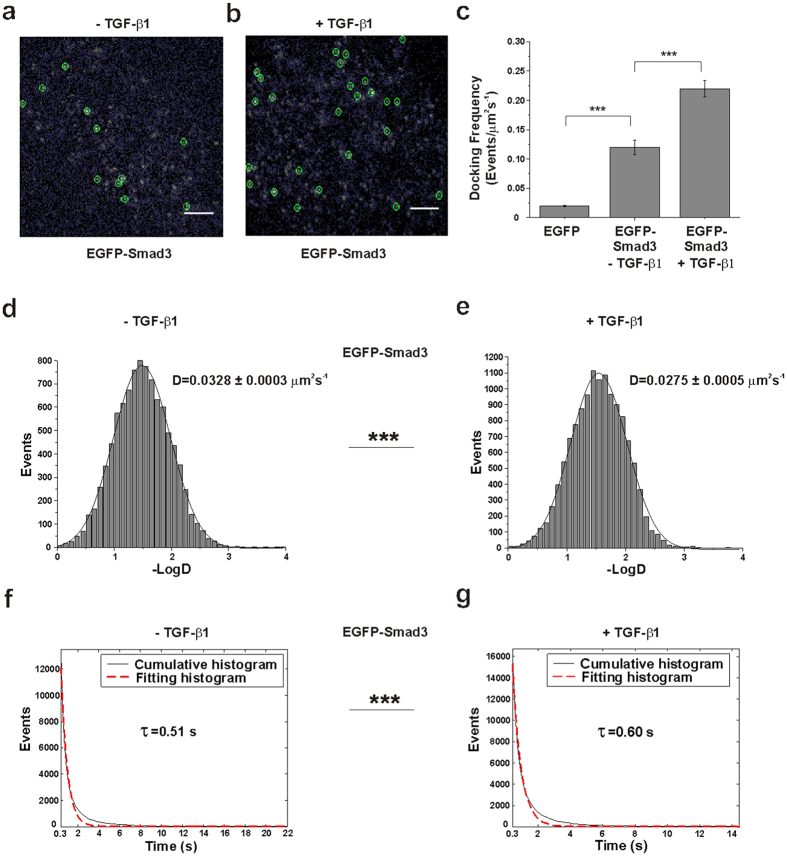
Total internal reflection fluorescence microscopy (TIRFM) imaging revealed membrane-docking behaviors of EGFP-Smad3 under different conditions. (**a,b**) Two typical single-molecule fluorescence images of individual EGFP-Smad3 molecules (enclosed in green circles) docking on the HeLa cell membrane in the absence (**a**) and presence (**b**) of transforming growth factor β1 (TGF-β1). Scale bars: 5 μm. (**c**) Comparison of the docking frequency of EGFP-Smad3 on the plasma membrane of HeLa cells before and after TGF-β11 treatment. The total number of single EGFP-Smad3 molecules docking on cell membrane was counted for 30 s and calculated as docking frequency per μm^2^ per s for every single cells. Data are shown as mean frequency ± SEM from 8 cells. The docking frequencies of EGFP-Smad3 were 0.12 ± 0.01 and 0.22 ± 0.01 events/μm^2^s^−1^ in the absence and presence of TGF-β1, respectively. The docking frequency of EGFP (0.020 ± 0.001 events/μm^2^s^−1^) was calculated as the control to exclude the signal from cytoplasm (***p < 0.001, t-test). (**d,e**) Diffusion rates (D) of membrane-docked EGFP-Smad3 molecules with the marked D value in HeLa cells in the absence (**d**) and presence (**e**) of TGF-β1 respectively. The two groups of D values are significantly different (p < 0.001, Mann-Whiteney U test). (**f,g**) Cumulative histograms (solid) indicated the on-times of EGFP-Smad3 molecules in unstimulated cells (**f**) and stimulated cells (**g**) respectively. The histograms were fitted with a single exponential function. The dotted lines are the fitting curves with the time constants τ_u_ (0.51 s) and τ_s_ (0.60 s). The correlation coefficients of the fitting lines were both above 0.97. The two groups of τ values are significantly different (p < 0.001, Mann-Whiteney U test).

HeLa cells expressing EGFP-Smad3 at a low expression level were used to study the docking of single Smad3 molecules to cell membrane. EGFP-Smad3 was phosphorylated and activated like endogenous Smad3 in response to TGF-β1 (Supplementary Figure 1). Single-molecule imaging of EGFP-Smad3 was firstly carried out in resting cells. As shown in Fig. 1a, a few Smad3 molecules were observed as well-dispersed diffraction-limited fluorescent spots on the cell membrane. For most spots, their fluorescence endured less than 2 s before suddenly disappeared (Supplementary movie 1). The fluorescence intensity distribution of these individual spots was similar to that of purified single EGFP molecules on coverslip (Supplementary Figure 2a,b), indicating they represented single EGFP-Smad3 molecules^23^,^27^.

Cells after TGF-β1 stimulation were then checked under the same conditions. Single-molecule imaging of membrane-docked Smad3 was also achieved, basing on the intensity distribution of the dispersed diffraction-limited fluorescent spots (Fig. 1b; Supplementary Figure 2c and Supplementary movie 2). It was clearly observed that more single Smad3 molecules docked to cell membrane. By calculating the docking frequency of EGFP-Smad3 molecules appeared on cell membrane during 30 seconds, we found the value was increased from 0.12 ± 0.01 to 0.22 ± 0.01 events/μm^2^s^−1^ after TGF-β1 stimulation (Fig. 1cp < 0.001). Docking of the control EGFP which was randomly diffused in the cytoplasm was hardly detected (0.020 ± 0.001 events/μm^2^s^−1^, Supplementary Figure 4b,g), demonstrating the possibility of imaging intracellular molecules accidentally moving through or near the evanescent field of TIRFM was very low in our measurement and could be ignored.

We also performed the ensemble measurement to confirm the ligand-induced increase of Smad3 docking with cells expressing a higher level of EGFP-Smad3. 70% of the cells showed a significant increase in fluorescence within 15 min after TGF-β1 stimulation (Supplementary Figure 3), whereas all the unstimulated cells exhibited a gradual fluorescence decrease with time because of photo-bleaching. As we know, the increase in fluorescence on the cell membrane was a sum of ensemble EGFP-Smad3 molecules with unsynchronized behaviors of membrane association and dissociation. Therefore, we mainly used single-molecule imaging of EGFP-Smad3 at low expression level in the following studies to investigate the membrane-docking dynamics of Smad3.

### Change in docking dynamics of Smad3 after stimulation

We characterized the docking behaviors of EGFP-Smad3 from time-lapse single-molecule images. Besides observing an increase in number of molecules docked on the cell membrane after TGF-β1 stimulation, we found the docking behaviors of EGFP-Smad3 were different in unstimulated and stimulated cells. Using a robust single-molecule tracking program (u-Track)^30^,^31^,^32^, we tracked the diffusion trajectories of thousands of individual EGFP-Smad3 molecules at the plasma membrane for further analysis. TGF-β1 stimulation shifted the diffusion coefficients (D) of Smad3 molecules toward lower values, from 0.0328 ± 0.0003 μm^2^s^−1^ in unstimulated cells to 0.0275 ± 0.0005 μm^2^s^−1^ in stimulated cells (Fig. 1 panels d and e, ***p < 0.001 for 2 groups of diffusion coefficients). Furthermore, we quantified the membrane-docking times of each EGFP-Smad3 molecules by measuring their fluorescence dwell times or on-times (starting from when the fluorescence signal appeared to when it disappeared). Cumulative histograms of the fluorescence on-times were fitted with a single exponential decay for both unstimulated and stimulated cells. We found a longer on-times with stimulated cells (0.60 s) than unstimulated cells (0.51s) (Fig. 1 panels f and g, ***p < 0.001 for 2 groups of on-times), indicating a longer membrane association time for EGFP-Smad3 in TGF-β1-stimulated cells than unstimulated cells.

Of note, the diffusion coefficient of EGFP-Smad3 was much higher than that of EGFP molecules fixed on coverslip (0.0036 ± 0.0001 μm^2^s^−1^, Supplementary Figure 2e), indicating the minimal contribution of the systematic error of microscopic drifting. Moreover, the dwell times with unstimulated and stimulated cells (τ_u_ and τ_s_) were much shorter than the photo-bleaching time of single EGFP molecules under the same excitation conditions (4.41 s) (Supplementary Figure 2d) and were unchanged when we increased the excitation power. Therefore, the disappearance of individual EGFP-Smad3 fluorescence signal was due to the dissociation of EGFP-Smad3 from cell membrane instead of EGFP photo-bleaching. Different docking characteristics of EGFP-Smad3 suggested different regulation mechanisms in unstimulated and stimulated cells.

### Binding of Smad3 with TRI for activation

**Figure 2:**
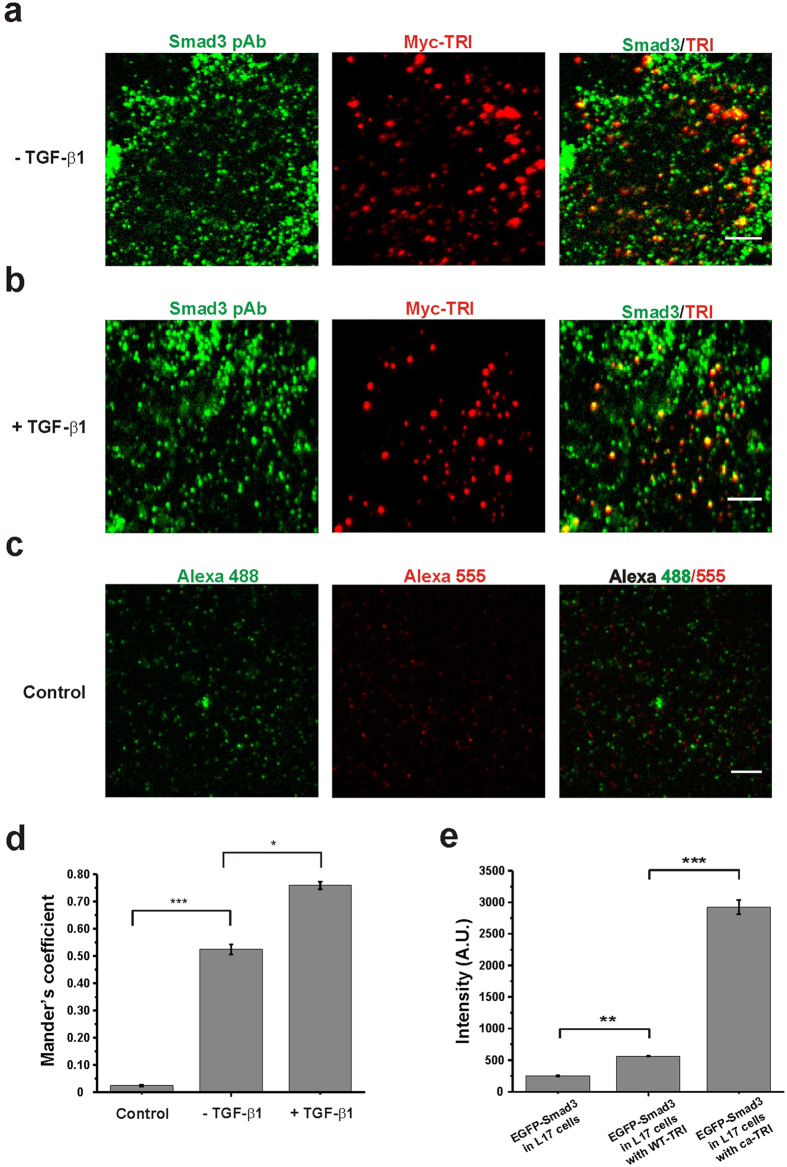
Binding of Smad3 with TRI on the cell membrane. (**a,b**) Dual-view TIRFM imaging of Smad3 (pAb, Alexa-488) and Myc-TRI (Myc mAb, Alexa-555) in the Myc-TRI-expressing HeLa cells before (**a**) and after (**b**) TGF-β1 stimulation. The cells were immunostained with antibodies against Myc and Smad3. Scale bars: 5 μm. (**c**) Dual-view TIRFM imaging of Alexa-488 and Alexa-555 in the Myc-TRI-expressing HeLa cells. The cells were immunostained with the secondary antibodies but without using the primary antibody. They were used to exclude the non-specific binding of antibodies and the cross-talk of the imaging setups. Scale bar: 5 μm. (**d**) Quantification of colocalization of Myc-TRI with Smad3 using the Mander’s coefficient (Blobprob plugin, ImageJ). 52.4% ± 1.9% (n = 6 cells; mean ± SEM) and 75.9% ± 1.4% (n = 6 cells; mean ± SEM) of TRI colocalized with Smad3 in the absence and presence of TGF-β1, respectively. And the colocalization coefficient of the control experiment was 2.5% ± 0.3% (n = 6 cells; mean ± SEM) (*p < 0.05 and ***p < 0.001, t-test). (**e**) The averaged fluorescence intensity of the EGFP-Smad3 molecules at high expressing level on the plasma membrane of TRI-deficient L17 cells, L17 cells expressing WT-TRI-HA and ca-TRI-HA were quantified and compared. Data are mean intensity ± SEM from 6 cells (**p < 0.01 and ***p < 0.001, t-test).

As Smad3 is activated by TRI during TGF-β signaling, we tested whether the membrane docking of Smad3 is dependent on TRI. Single-molecule fluorescence imaging of the transfected EGFP-Smad3 was examined in R1B/L17 cells, which have undetectable endogenous expression of TRI (Supplementary Figure 4a)^33^,^34^. We found that in those cells, membrane docking of Smad3 was inhibited as the docking frequency significantly decreased to a value (0.018 ± 0.001 events/μm^2^s^−1^) similar to the random membrane-association of the control intracellular protein (Supplementary Figure 4g). We then co-transfected EGFP-Smad3 into the L17 cells with wild-type TRI (WT-TRI) or constitutively activated TRI (ca-TRI) for ensemble Smad3 measurement. The fluorescence signal from EGFP-Smad3 on the cell membrane was obviously increased in the L17 cells expressing WT-TRI, and it became even higher with ca-TRI-expressing L17 cells (Supplementary Figure 4c–e and Fig. 2e). Therefore, the membrane docking of Smad3 was depending on TRI even in unstimulated cells, and was enhanced with the activated TRI.

To further verify the direct interaction of Smad3 with TRI on the cell membrane, we transfected HeLa cells with Myc-tagged TRI and used TIRFM to image the antibodies labeled endogenous Smad3 and Myc-tagged TRI on the plasma membrane. Smad3 and Myc-TRI were colocalized both in unstimulated and stimulated HeLa cells, and a higher degree of colocalization was found after TGF-β1 treatment (52.4% ± 1.9% and 75.9% ± 1.4% of TRI colocalized with Smad3 in the absence and presence of TGF-β1 respectively, Fig. 2 panels a, b and d, *p < 0.05). As a control, HeLa cells expressing Myc-TRI were only immunostained with the secodary antibodies to check the colocalization (2.5% ± 0.3%, Fig. 2c) and ensure the non-specific binding of the antibodies and cross-talk of the imaging setups were not a problem.

### Activation of the docked Smad3 molecules on membrane

**Figure 3:**
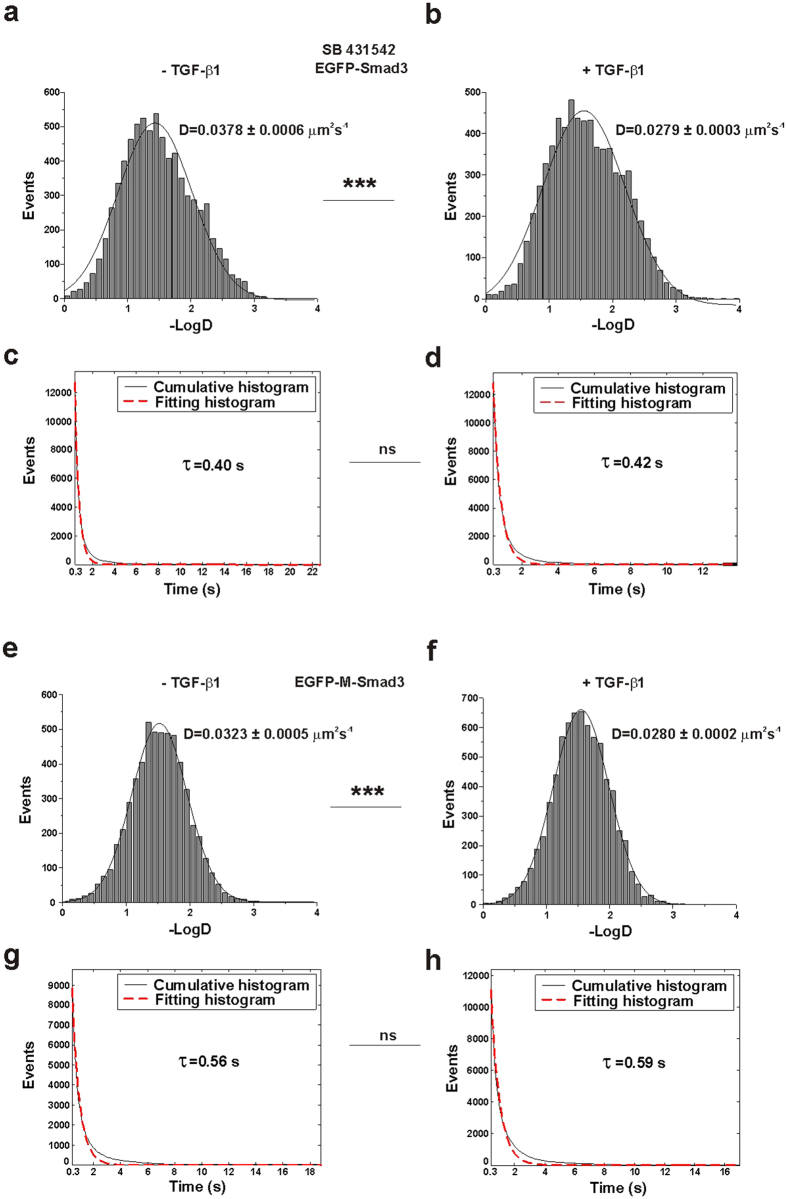
Regulation of Smad3 membrane docking under different conditions. (**a,b**) Diffusion rates (D) of membrane-docked EGFP-Smad3 molecules in the SB431542-pretreated HeLa cells with the marked D value before (**a**) and after (**b**) TGF-β1 stimulation. The two groups of D values are significantly different (p < 0.001, Mann-Whiteney U test). (**c,d**) Cumulative histograms (solid) for the on-times of EGFP-Smad3 molecules in SB431542-pretreated HeLa cells before (**c**) and after (**d**) TGF-β1 stimulation. The histograms were fitted with a single exponential function. The dotted lines are the fitting curves with the time constants τ_u_ (0.40 s) and τ_s_ (0.42 s). The correlation coefficients of the fitting lines were both above 0.97. The two groups of τ values are not significantly different (p > 0.05, Mann-Whiteney U test). (**e,f**) Diffusion rates (D) of membrane-docked EGFP-M-Smad3 molecules in HeLa cells with the marked D value before (**e**) and after (**f**) TGF-β1 stimulation. The two groups of D values are significantly different (p < 0.001, Mann-Whiteney U test). (**g,h**) Cumulative histograms (solid) for the on-times of EGFP-M-Smad3 molecules in HeLa cells before (**g**) and after (**h**) TGF-β1 stimulation. The histograms were fitted with a single exponential function. The dotted lines are the fitting curves with the time constants τ_u_ (0.56 s) and τ_s_ (0.59 s). The correlation coefficients of the fitting lines were both above 0.97. The two groups of τ values are not significantly different (p > 0.05, Mann-Whiteney U test).

The above results indicated that Smad3 binds to TRI on the cell membrane in both resting and stimulated cells, but the membrane docking dynamics of Smad3 are different. We sought to examine the relationship between the docking dynamics and activation of Smad3. We pretreated the cells with SB431542, which can inhibit the phosphorylation of TRI^35^. According to our single-molecule tracking results (Supplementary movie 3), while TGF-β1 stimulation still shifted the diffusion coefficients (D) of Smad3 molecules toward lower values, from 0.0378 ± 0.0006 μm^2^s^−1^ in unstimulated cells to 0.0279 ± 0.0003 μm^2^s^−1^ in stimulated cells (Fig. 3 panels a and b, ***p < 0.001 for 2 groups of diffusion coefficients), the membrane-docking times were unchanged (0.40 s vs. 0.42 s, Fig. 3 panels c and d, p > 0.05 for 2 groups of on-times).

It is known that activation of Smad3 is realized by the phosphorylation of its C-terminal SSXS motif^36^,^37^,^38^. We then constructed an EGFP-labeled mutated Smad3 (EGFP-M-Smad3) with the SSXS motif deleted to prevent its phosphorylation but keep its TRI-binding capability. We compared the diffusion coefficient (D) and the dwell time (τ) of EGFP-M-Smad3 on the cell membrane before and after TGF-β1 treatment (Supplementary movie 4). Similar results were obtained that the EGFP-M-Smad3 molecules showed slower diffusion rates in stimulated cells (decreased to 0.0280 ± 0.0002 μm^2^s^−1^ in stimulated cells from 0.0323 ± 0.0005 μm^2^s^−1^ in unstimulated cells, Fig. 3 panels e and f, ***p < 0.001 for 2 groups of diffusion coefficients), but no significant change in the membrane-docking dwell times (0.56 s vs. 0.59 s, Fig. 3 panels g and h, p > 0.05 for 2 groups of on-times). From the structure of Smad3, its receptor-binding domain (the L3 loop within the MH2 domain) is separated from its phosphorylation domain (C-terminal serine-rich SSXS motif)^39^. Thus, after stimulation, the membrane-docked M-Smad3 showed a similar decrease in membrane diffusion rate as the wild-type Smad3 for receptor binding but an unchanged membrane dissociation rate because of inefficient phosphorylation.

Therefore, it is expected that the ligand-induced Smad3 activation accounts for the change in its membrane docking dynamics. In the stimulated cells, while the decreased diffusion of TRI-bound Smad3 is due to the formation of heteromeric TRI/TRII signaling complex, the longer docking time of Smad3 suggests a more stable binding of Smad3 to TRI for Smad3 phosphorylation and activation.

**Figure 4:**
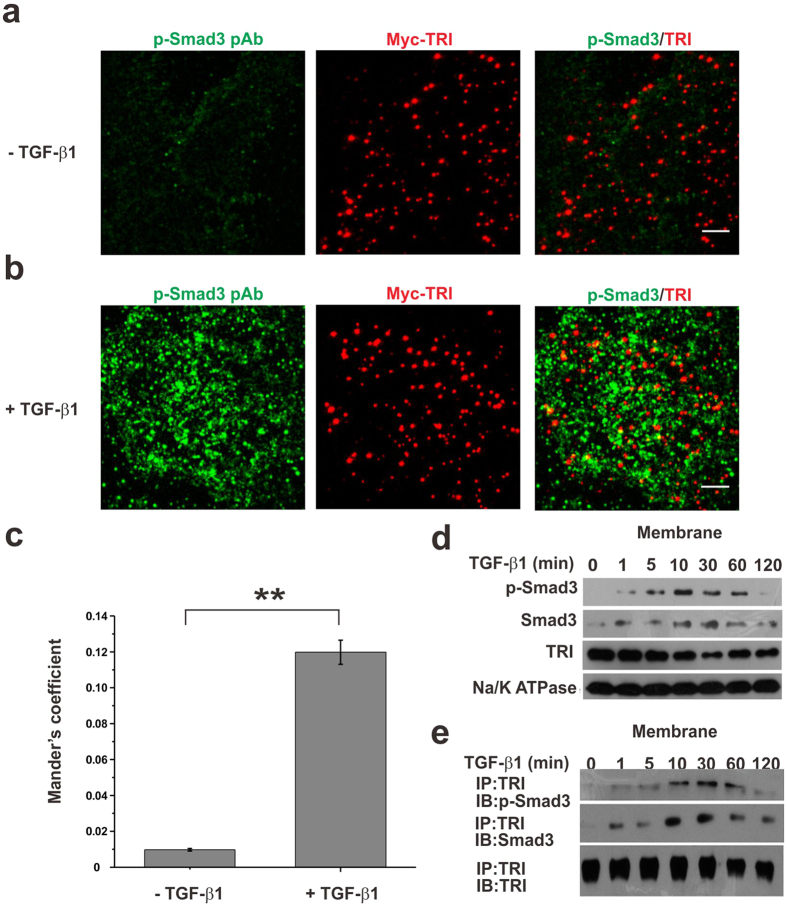
TGF-β1 induced Smad3 docking to membrane for activation. (**a,b**) Dual-view TIRFM images of p-Smad3 (pAb, Alexa-488) and Myc-TRI (Myc mAb, Alexa-555) before (**a**) and after (**b**) TGF-β1 stimulation in the Myc-TRI expressing HeLa cells. The cells were immunostained with antibodies against Myc and p-Smad3. Scale bars: 5 μm. (**c**) Quantification of colocalization of p-Smad3 with Myc-TRI using the Mander’s coefficient (Blobprob plugin, ImageJ). 0.98% ± 0.07% (n = 6 cells; mean ± SEM) and 11.98% ± 0.67% (n = 6 cells; mean ± SEM) of p-Smad3 molecules were colocalized with Myc-TRI in the absence and presence of TGF-β1, respectively. (**p < 0.01, t-test). (**d**) Western blotting analyses of p-Smad3, Smad3 and TRI. HeLa cells were treated with 10 ng/ml TGF-β1, harvested at the indicated time points (0, 1, 5, 10, 30, 60, 120 min), and then subjected for western blotting analyses of p-Smad3, Smad3 and TRI in the membrane extraction. Na/K ATPase was used as the internal control for membrane extraction. (**e**) Co-IP analysis of the interaction between TRI and Smad3 or p-Smad3 using the membrane fractions from HeLa cells. The cells were treated with 10 ng/ml TGF-β1, harvested at the indicated time points (0, 1, 5, 10, 30, 60, 120 min), and subjected for co-IP analysis.

Other biochemical experiments were carried out to confirm that Smad3 is activated on cell membrane. From the dual-view TIRFM imaging of phosphorylated Smad3 (p-Smad3) and TRI, while very few p-Smad3 molecules were detected on the membrane of resting cells which was possibly due to the secreted of TGF-β1 by HeLa cells themselves, they were clearly observed on the cell membrane in presence of TGF-β1 and higher degree of colocalization with TRI was found after TGF-β1 treatment (from 0.98% ± 0.07% to 11.98% ± 0.67%, Fig. 4 panels a–c). The colocalization frequency of p-Smad3 and TRI after TGF-β1 treatment is also much higher than that of random colocalization (2.5% ± 0.3%, Fig. 2c).

We then separated total cell proteins as those in membrane fractions and cytosol fractions, and checked the phosphorylation level of Smad3 using Western blot analysis. In the resting cells, p-Smad3 could not be detected in the membrane fractions, but it was clearly observed after cells were incubated with TGF-β1 for 5 min. Meanwhile, the total expression level of Smad3 in the cells stimulated by TGF-β1 was not changed (Fig. 4d). We also used anti-TRI antibody to pull down the proteins interacted with TRI. p-Smad3 could be detected in the membrane fractions of the stimulated cells, but not in that of the resting cells (Fig. 4e). All above results demonstrated that phosphorylation of Smad3 could take place at cell membrane. Therefore, in response to TGF-β1 stimulation, Smad3 molecules interacted with TRI for a longer time for its phosphorylation and activation.

### Role of endocytosis in Smad3 activation

**Figure 5:**
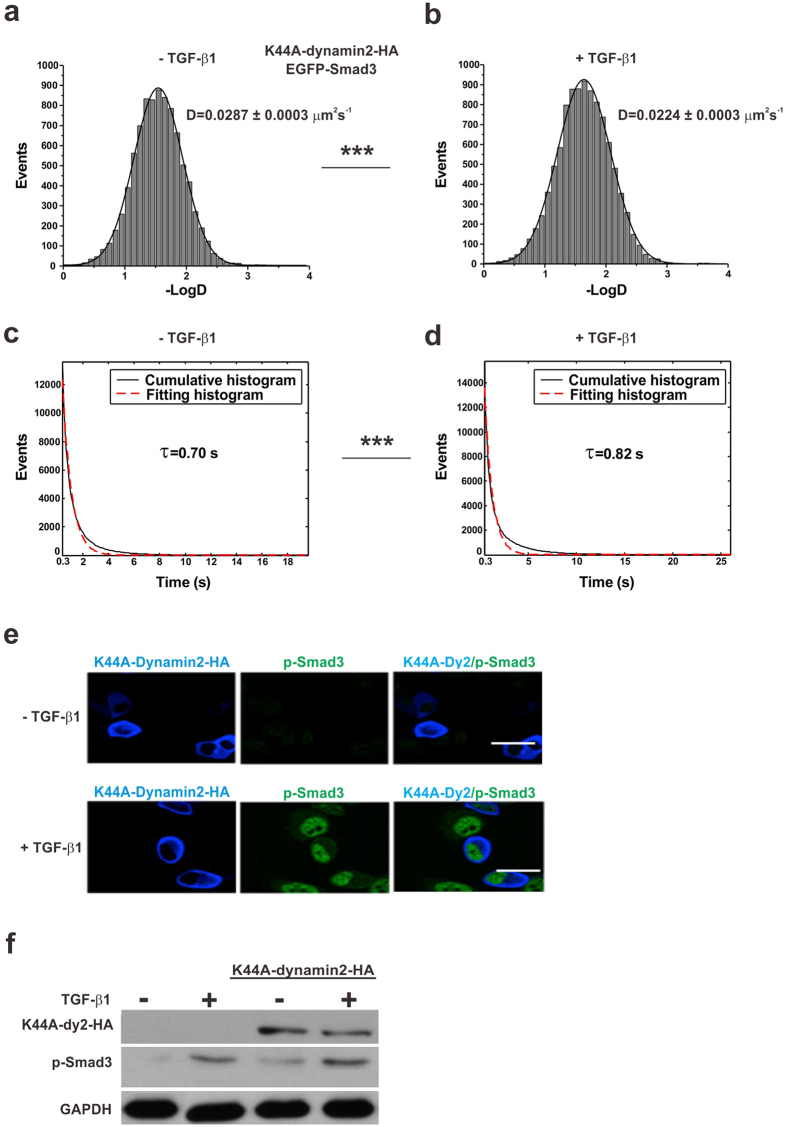
Membrane docking of Smad3 is endocytosis-independent. (**a,b**) Diffusion rates (D) of membrane-docked EGFP-Smad3 molecules with the marked D value before (**a**) and after (**b**) TGF-β1 stimulation in HeLa cells expressing K44A-dynamin2-HA to inhibit endocytosis. The two groups of D values are significantly different (p < 0.001, Mann-Whiteney U test). (**c,d**) Cumulative histograms (solid) of the on-times of EGFP-Smad3 molecules before (**c**) and after (**d**) TGF-β1 stimulation in HeLa cells expressing K44A-dynamin2-HA to inhibit endocytosis. The histograms were fitted with a single exponential function. The dotted lines are the fitting curves with the time constants τ_u_ (0.70 s) and τ_s_ (0.82 s). The correlation coefficients of the fitting lines were both above 0.97. The two groups of τ values are significantly different (p < 0.001, Mann-Whiteney U test). (**e**) The effect of endocytosis on Smad3 phosphorylation and nuclear transportation shown by confocal microscopy. HeLa cells expressing K44A-dynamin2-HA were immunostained with antibodies against HA and p-Smad3. Confocal imaging of p-Smad3 (pAb, Alexa-488) and K44A-dynamin2-HA (HA mAb, Alexa-647) before and after TGF-β1 stimulation indicated that the nuclear accumulation of p-Smad3 was not dependent on endocytosis. Scale bars: 5 μm. (**f**) Western blotting of K44A-dynamin2-HA and p-Smad3. GAPDH was used as the internal control.

Ligand–receptor binding is known to trigger the internalization of the TGF-β signaling complex into cytoplasm^14^,^40^,^41^,^42^. By checking the phosphorylation and nuclear accumulation of R-Smads in the cells pretreated with endocytosis inhibition reagents, previous studies, even with similar experimental systems, have yielded controversial results regarding the dependence of TGF-β signaling and R-Smads activation on endocytosis^14^,^43^. To investigate the role of endocytosis on Smad3 activation with live cells, we first analyzed single-molecule images of EGFP-Smad3 using cells expressing mutant dynamin (K44A-dynamin2-HA) to block endocytosis (Supplementary Figure 5a and Supplementary movie 5). The membrane diffusion dynamics of EGFP-Smad3 in cells with endocytosis inhibition were consistent with that of cells without endocytosis inhibition. TGF-β1 stimulation shifted the diffusion coefficients (D) of Smad3 molecules toward lower values, from 0.0287 ± 0.0003 μm^2^s^−1^ in unstimulated cells to 0.0224 ± 0.0003 μm^2^s^−1^ in stimulated cells (Fig. 5 panels a and b, ***p < 0.001 for 2 groups of diffusion coefficients). And we also found a longer docking time with stimulated cells (0.82 s) than unstimulated cells (0.70 s) (Fig. 5 panels c and d, ***p < 0.001 for 2 groups of on-times). The results from single-molecule imaging in living cells provided the evidence of Smad3 activation in the endocytosis-blocking cells. Moreover, fluorescence imaging and western blot of the cells also showed that both the phosphorylation and the nuclear accumulation of Smad3 were independent on endocytosis (Fig. 5 panels e and f).

More reagents with different inhibition mechanisms were used to examine effect of endocytosis on Smad3 activation. These include dynasore to inhibit dynamin-dependent endocytic pathways^44^,^45^ and clathrin siRNA to dampen clathrin-dependent endocytosis^46^. The same results were obtained that neither phosphorylation nor the nuclear accumulation of Smad3 was affected (Supplementary Figure 6).

In addition, we used the method of potassium depletion which is known to inhibit the internalization of various receptors, including TGF-β receptors^14^,^40^. From the immunofluorescence results, we found that both the clathrin and caveolin-1 dependent endocytic pathways were blocked (Supplementary Figure 5b). When we checked the single-molecule images of EGFP-Smad3 in cells treated by potassium depletion medium, we found more EGFP-Smad3 molecules were docked to the cell membrane after potassium depletion (Supplementary Figure 5d,e). This is because more TRI molecules were available for Smad3 binding on the cell membrane after the inhibition of TRI endocytosis. Western blot analysis indicated that the levels of p-Smad3 in the membrane protein extraction increased after stimulating the potassium-depleted cells by TGF-β1 (Supplementary Figure 5c). All these evidences together strongly suggest that the activation of Smad3 at the cell membrane is independent on endocytosis.

## Discussion

Smad3 is a key signal transducer in TGF-β signaling, and potential therapeutic target in TGF-β signaling involved diseases. Many previous studies proposed that R-Smads transduce the TGF-β signal in early endosomes, which is believed to be a compartment specialized for assembling and propagating signals^10^,^17^,^42^,^43^. However, a few studies reported that after ligand stimulation R-Smads were recruited to the plasma membrane based on indirect evidences from the co-IP of R-Smads and TGF-β receptors in the endocytosis-blocking cells^14^,^15^,^16^. In this work, we directly observed the membrane docking of single Smad3 molecules in living cells under both unstimulated and stimulated conditions by using the advanced method of single-molecule imaging and tracking. This enables the quantification of different membrane-docking dynamics, which reflects the regulation of Smad3 for activation.

Previously, it has been reported that the complex of Smad3 and TGF-β receptor could be formed at the cell membrane, then the whole complex internalized into early endosomes to induce Smad3 phosphorylation and downstream signaling^15^,^16^. This situation did not likely occur in our system because there was no significant difference in membrane diffusion dynamics of single Smad3 molecules in the cells with and without endocytosis. Of note, since our single-molecule TIRFM images focus only on the cell membrane, the possibility that the intracellular Smad3 may also be activated by the internalized TGF-β receptor in early endosomes cannot be excluded.

**Figure 6:**
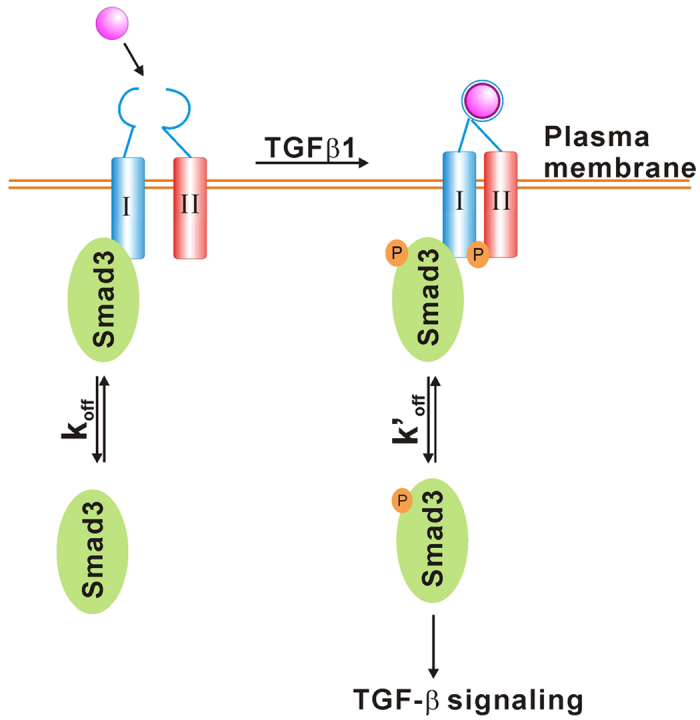
A model for Smad3 activation at the cell membrane. See Discussion for details.

Besides the quantitative data from single-molecule imaging, other *in vitro* biochemical assays including colocalization imaging, western blot and pull-down experiments all supported our discovery. Based on these results, we propose a model depicting Smad3 activation at the cell surface (Fig. 6). In the absence of stimulus, Smad3 molecules are shuttled between cytosol and cell membrane, and they can associate with TRI, but this association is less stable with a dissociation rate constant (*k*_*off*_) of 1.96 s^−1^ (1/τ_u_). Once the cell perceives stimuli, such as TGF-β1, Smad3 binds to the activated TGF-β1/TRI/TRII complex for phosphorylation at the cell membrane. The phosphorylation of Smad3 leads to the longer receptor binding time (*k′*_*off*_ 1.67 s^−1^) before the shuttling of Smad3 to cytoplasm for signaling.

In summary, by monitoring the change in membrane docking dynamics of Smad3 with live-cell single-molecule fluorescence microscopy and single molecule tracking methods, we have demonstrated that Smad3 could be phosphorylated and activated on the cell membrane through binding with TRI. This result provides new information on the regulation of Smad3 activation, and offers a new approach to investigate the intracellular signaling proteins for better understanding their functions in signal transduction.

## Methods

### Cell culture

HeLa cells were cultured in Dulbecco’s modified Eagle’s medium (DMEM, Gibco) while the TRI-deficient mink lung epithelial L17 cells^33^,^34^ were maintained in minimum essential medium (MEM Gibco), supplemented with 10% fetal bovine serum (Hyclone) and antibiotics (50 mg/mL streptomycin, 50 U/mL penicillin) at 37 °C in a 5% CO_2_ atmosphere. HeLa cells were used for most experiments unless specified.

### Plasmids, transfection and drug treatment

Full-length human Smad3 cDNA was amplified from pCMV5B-Smad3 vector^47^. The mutant Smad3 (M-Smad3) was constructed by deleting the sequence that codes the COOH-terminal SSXS motif of Smad3. Smad3 and M-Smad3 sequences were ligated into the pEGFP-C1 construct (Clonetech) to generate the EGFP-Smad3 and EGFP-M-Smad3 plasmids, respectively. Plasmid of Myc-TRI was constructed as previously described^48^. Plasmid encoding K44A-dynamin2-HA was a generous gift from Prof. Youyi Zhang (Peking University, China)^49^. Transfection was performed using Lipofectamine 2000 (Invitrogen) according to the manufacturer’s instructions.

For imaging EGFP-Smad3 or EGFP-M-Smad3 at the single-molecule level, cells grown in a 35-mm glass-bottom dish (Shengyou Biotech, China) were transfected with 0.3 μg/ml EGFP-Smad3 or EGFP-M-Smad3 plasmid for 5 hours, washed, then imaged in phenol red-free DMEM. For imaging ensemble Smad3 molecules, cells were serum-starved in medium containing the EGFP-Smad3 plasmid for the first 5 hours, then washed, changed to complete DMEM with serum for another 5 hours to increase protein expression, and then underwent fluorescence imaging in phenol red-free DMEM. For imaging EGFP-Smad3 in living cells without receptor endocytosis, cells were transfected with 2 μg/ml K44A-dynamin2-HA for 24 hours before EGFP-Smad3 transfection.

For imaging stimulated cells, cells transfected with EGFP-Smad3 or EGFP-M-Smad3 were treated with 10 ng/ml TGF-β1 (R&D, USA) in phenol red-free DMEM for 15 min at 37 °C before fluorescence imaging. For experiments involving SB431542, the transfected cells were incubated with 20 μM SB431542 (Sigma) for 2 hours, followed by the addition of 10 ng/ml TGF-β1 for 15 min, and fluorescence imaging at 37 °C.

### Antibodies and reagents

Antibodies against Myc (9E10, 1:200), HA (Y-11, 1:100), TRI (V-22, 1:200) were obtained from Santa Cruz Biotechnology. Antibody against clathrin (1:200) was from BD bioscience. Antibodies against Smad3 (1:200) and p-Smad3 (1:200) were from Cell Signaling Technology. Mouse and rabbit Alexa Fluor 488-, 555- and 647- conjugated secondary antibodies (1:500), Aleax Fluor 488- and 568-conjugated transferrin (20 μg/mL) and Aleax Fluor 555-conjugated Cholera Toxin B (CTB-555 20 μg/mL) were all from Molecular Probes. SB431542 (20 μM) was from Sigma. Dynasore (80–160 μM) was from Selleckchem. Human TGFβ1 (10 ng/mL) was from R&D.

### Single-molecule fluorescence imaging

Single-molecule fluorescence measurements were performed on a home-built objective-type total internal reflection fluorescence microscope (TIRFM) which was based on Olympus IX71 inverted microscope^23^,^27^. Briefly, two solid lasers were used to excite the fluorophores (EGFP, Alexa Fluor 488 and Alexa Fluor 555). The fluorescence was collected through an oil-immersion objective (100x, 1.45NA, Olympus), separated spectrally with a dual-view assembly (Optical insights), and properly filtered with two band-pass filters (525/50 for EGFP and Alexa Fluor 488 and 617/73 for Alexa Fluor 555) before being projected onto a 14-bit back-illuminated electron-multiplying charge-coupled camera (EMCCD, Andor Technology DU-897D-BV). The notch filter (NF01-488 and NF01-568, Semrock) was also applied to avoid scattered laser light. The typical excitation power measured after the objective in epi-mode was fixed to 1 mW for live cell imaging. Movies of 300 frames were acquired for each sample at a frame rate of 10 Hz. Alternating laser excitation between two wavelengths was employed to check the colocalization of Smad3 and TRI at cell membrane and the instrument was operated with two mechanical shutters (Vincent Associates, Uniblitz LS3Z2-100) synchronized with the EMCCD. Image acquisition was controlled by Andor iQ software and a z-axis negative feedback equipment (MFC-2000).

For the control experiment of single EGFP molecule imaging on coverslips, active EGFP protein (Abcam) was immobilized on the coverslips through biotin coupled EGFP antibody (Invitrogen) as previously reported^27^.

### Single-molecule detection and tracking

Time-lapse image sequences were acquired and then tracked with u-Track methods as described^31^. By fitting Gaussian kernels, approximating the two-dimensional point spread function of the microscope around local intensity maxima, the sub-pixel localization was achieved for detection of individual spots. To construct the intact trajectories, the algorithm first links the detected spots between consecutive frames, and then links the track segments generated to close gaps and capture particle merge and split events.

### Analysis of the dynamics of single molecules docked on cell membrane

From the time-lapse TIRFM images, only fluorescent trajectories lasting more than five consecutive frames were collected for 2D-mean square displacement (MSD) calculation for the sake of precise fitting. 2D-MSD was calculated for each time interval Δt (Δt = nδt, with δt = 100 ms) over a trajectory with the following equation^50^,^51^.
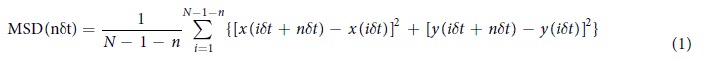
where (x(iδt + nδt), y(iδt + nδt)) is the spot position at time interval Δt after starting at position (x(iδt), y(iδt)); N is the total number of image frames before the molecule disappeared; and n and i are integers, with n determining the time increment.

The diffusion coefficient D was calculated from the slope of the first 4 points (100 ms) in each MSD–Δt plot by least-square fitting according to MSD = 4DΔt. D distribution histograms were obtained under different conditions. Under each condition, we repeated the experiment three times to obtain three histograms of diffusion coefficient, and each histogram represented the data from about ten thousands single Smad3 molecules in 8 cells. The most probable D values were obtained by fitting each histogram with Gaussian distribution, and the D value was shown as mean ± SEM (n = 3).

### Measurement of on-times and kinetics of single molecules docked on cell membrane

Starting from a membrane-dissociation state, the membrane-association state was defined as when the fluorescence intensity increased to >3-fold the standard deviation above the mean for longer than 2 frames^29^,^52^. Each period of association was defined as an on-time. Each test involved above ten thousands of association events from 8 cells. Cumulative histograms of on-times were used to avoid the effect of bin size on the kinetic parameters. The histograms were fitted to a single exponential function by use of Matlab. The single exponential function is described as
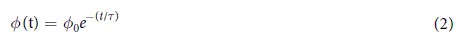
where *ϕ*(*t*) is the on-time function, *ϕ*_0_ is a prefactor, and *τ* is the time constant.

### Dual-view TIRFM imaging for studying co-localization at the cell membrane

Live-cells labeling for colocalization imaging was carried out as described^41^. Briefly, cells expressing Myc-TRI were sequentially incubated with anti-Myc antibody and the fluorescent-conjugated secondary antibody at 4 °C for 1 hour. Then the cells were washed with PBS for 2 times, permeabilized with PBS containing 0.5% Triton X-100 for 10 min, and then fixed with 4% formaldehyde in PBS for 20 min at room temperature. Then the cells were washed with PBS for 3 times and incubated in the blocking buffer (PBS, 0.5% bovine serum albumin) for 30 min. After that, the cells were incubated with the diluted primary antibodies (anti-Smad3 or anti-p-Smad3) in blocking buffer overnight at 4 °C, washed with PBS for 3 times, and then incubated with the diluted fluorescent conjugated secondary antibodies in blocking buffer for 1 hour at 4 °C. The signals were detected with TIRFM described above. Colocalization of Myc-TRI and Smad3 was determined by using the Blobprob ImageJ plugin as previously reported^53^. We found the Mander’s coefficients determined by Blobprob ImageJ plugin matched well with the colocalization level determined by visual inspection.

### Immunofluorescence

For immunostaining of intracellular molecules, cells were washed with PBS for 2 times, permeabilized with PBS containing 0.5% Triton X-100 for 10 min, and then fixed with 4% formaldehyde in PBS for 20 min at room temperature. Then the cells were washed with PBS for 3 times and incubated in the blocking buffer (PBS, 0.5% bovine serum albumin) for 30 min. After that, the cells were incubated with the diluted primary antibodies in blocking buffer overnight at 4 °C, washed with PBS for 3 times, and then incubated with the diluted fluorescent conjugated secondary antibodies in blocking buffer for 1 hour at 4 °C. For two or more molecules labeling, the procedures were repeated.

The prepared cell samples were imaged with a confocal microscope (FluoView FV1000-IX81, Olympus, Japan) equipped with a 100x, 1.40NA objective. For multi-color imaging, Aleax Fluor 488 was excited by a 488 nm laser (FV5-LAMAR, Olympus) and the emission was collected using a 500–545 nm band-pass filter. Aleax Fluor 555 and 568 were excited with by a 559 nm laser (FV10-LD550, Olympus) and the emission was collected using a 570–625 nm band-pass filter. Aleax Fluor 647 was excited with by a 635 nm laser (FV10-LD635, Olympus) and the emission was collected using a 655–755 nm band-pass filter. The fluorophores were excited by using the corresponding emission filters sequentially. In our experiment, the color bleed-through between different channels is negligible^22^.

### Statistics analysis

For robust single-molecule analysis, the Non-parametric Mann-Whiteney U test used in statistical analysis was performed with GraphPad Prism (GraphPad Software). P-value less than 0.05 were regarded as statistically significant. For the other statistical analysis using small sample size, the Student’s two-tailed t-test was performed with Origin 8.1 (OriginLab). P-value less than 0.05 were regarded as statistically significant.

## Additional Information

**How to cite this article**: Li, N. *et al*. Single-Molecule Imaging Reveals the Activation Dynamics of Intracellular Protein Smad3 on Cell Membrane. *Sci. Rep.* **6**, 33469; doi: 10.1038/srep33469 (2016).

## Supplementary Material

Supplementary Information

Supplementary Movie 1

Supplementary Movie 2

Supplementary Movie 3

Supplementary Movie 4

Supplementary Movie 5
