# Supplementary material for: Single-Molecule Imaging Reveals the Activation Dynamics of Intracellular Protein Smad3 on Cell Membrane
Source: Sci Rep. 2016 Sep 19;6:33469. doi: 10.1038/srep33469 (PMC5027577; doi:10.1038/srep33469)
Supplement: Supplementary Information [file srep33469-s1.pdf]

Supplementary Information

Single-Molecule Imaging Reveals the Activation Dynamics of Intracellular Protein Smad3 on Cell Membrane

Nan Li[1], Yong Yang[1], Kangmin He[1], Fayun Zhang[2], Libo Zhao[1], Wei Zhou[1], Jinghe Yuan[1], Wei Liang[2*], Xiaohong Fang[1*]

1 Beijing National Laboratory for Molecular Sciences, Institute of Chemistry, Key Laboratory of Molecular Nanostructure and Nanotechnology, Chinese Academy of Sciences, Beijing 100190, P. R. China.

2 Key Laboratory of Protein and Peptide Drugs, Institute of Biophysics, Chinese Academy of Sciences, Beijing 100101, P. R. China.

* To whom correspondence should be addressed: Xiaohong Fang, E-mail: xfang@iccas.ac.cn, Tel: 86-62653083; Wei Liang, Email: weixx@ibp.ac.cn.

Supplementary Results

Supplementary Methods:

Plasmid construction. Full-length human Smad3 cDNA was amplified by use of the FastStart High Fidelity PCR system (Roche) from pCMV5B-Smad3 vector. The two PCR primers included the sequences of EcoRI and XhoI restrictive endonucleases. The product of PCR was digested with EcoRI and XhoI, and then ligated with pEGFP-C1, which had been digested with EcoRI and XhoI. The *E. coli* strain BL21 was transformed with the ligation mixture. Then transformation colonies were chosen, and the recombinant plasmid (EGFP-Smad3) was extracted. After verification via PCR, restrictive endonuclease digestion and DNA sequencing, the recombinant plasmid was purified by using of the Endo Free Plasmid Maxi Kit (QIAGEN). The dominant-negative COOH-terminally truncated version of Smad3 (M-Smad3) was created by deleting the sequence coding for the COOH-terminal SSXS motif of Smad3. The M-Smad3 sequence was ligated into the pEGFP-C1 construct with EcoRI and XhoI digestion sites. The constructed plasmid was verified and used to transfect cells to express the EGFP-labeled mutant Smad3, named EGFP-M-Smad3.

Imaging analysis to identify single molecules. The central quarter of the EMCCD chip (256×256 pixel^2) was used for imaging analysis to ensure homogeneous illumination. The background fluorescence was first subtracted from each frame with the use of Image J software (NIH). Then every frame of each movie was used for selecting single-molecule fluorescent spots. The image was thresholded (5 times the mean intensity of an area without fluorescent spots) and filtered again with the use of a user-defined program in Matlab (Mathworks Corp.) to remove discrete signals. After the imaging process, the brightest pixel of each fluorescent spot within diffraction-limited size (3×3 pixels, 480×480 nm) was determined as the central position and enclosed with green circles. The spot with a peak pixel close to that of another spot (<3 pixels) was excluded.

Potassium depletion and membrane protein extraction. Endocytosis inhibition by potassium depletion was achieved as previously described[1]. Briefly, cells were incubated in DMEM: water (1:1) for 5 min at 37ºC followed by incubation in a minimal media (20 mM Hepes at pH 7.5, 140 mM sodium chloride, 1 mM calcium chloride, 1 mM magnesium sulphate, 5.5 mM glucose and 0.5 % BSA) for 1 hour at 37 ºC. Control cells were incubated in the minimal media supplemented with 10 mM potassium chloride. Cells were then harvested and proteins from the membrane and cytosol were extracted using Eukaryotic Membrane Protein Extraction Reagent kit (Perice). Lysates were then provided for Western blot analysis. The fractions from membrane were concentrated as 1/5 volume using ultrafiltration tube (10 kD, Millipore).

RNA interference of Clathrin. siRNA targeting clathrin heavy chain was purchased from GenePharma. The Sequences of siRNAs were as follows[2]: sense 5'-GUAAUCCAAUUCGAAGACCTT-3', anti-sense 5'-GGUCUUCGAAUUGGAUUACTT-3', and the non-sense sequence was: 5'-UUCUCCGAACGUGUCACGUTT-3'. The RNA interference was achieved by the transfection of 2 μg siRNA and 5 μL Lipofectamine 2000 (Invitrogen) for 24 - 36 hours, and was validated by immunofluorescence assays.

Western blotting. For western blotting, the cells were lysed in the assay buffer (50 mM Tris-HCl, pH 8.0, 150 mM NaCl, protease inhibitors), and the protein concentration in the lysates was determined by a spectrophotometer. Equal amount of the lysates were subjected to SDS-polyacrylamide gel electrophoresis (PAGE) and the immunoblotting was performed with primary antibodies and secondary antibodies conjugated to horseradish peroxidase (Abcam). Proteins were visualized by chemiluminescence. The signal density of phosphorylated proteins was normalized to the corresponding unphosphorylated proteins.

Co-Immunoprecipitation. HeLa cells were incubated with 10 ng/ml TGF-β1 for different times (0, 1, 5, 10, 30, 60, 120 min) after starved with serum free medium overnight. Cells were harvested and proteins from the membrane and cytosol were extracted using Eukaryotic Membrane Protein Extraction Reagent kit. Immunoprecipitation was performed by standard methodologies. Briefly, 2 μg rabbit polyclonal anti-TRI antibody (Santa Cruz Biotechnology) and 2 mg suspension were incubated for 4 hours, and then Agarose protein A/G beads were added for precipitation at 4 °C overnight. Precipitates were harvested by centrifuge at 4000 g/s for 3 min. After extensive washing, precipitates were boiled in lysis buffer for 10 min and then subjected to Western blotting analyses for detection of potential interacting proteins. Normal rabbit Ig G served as a negative control.

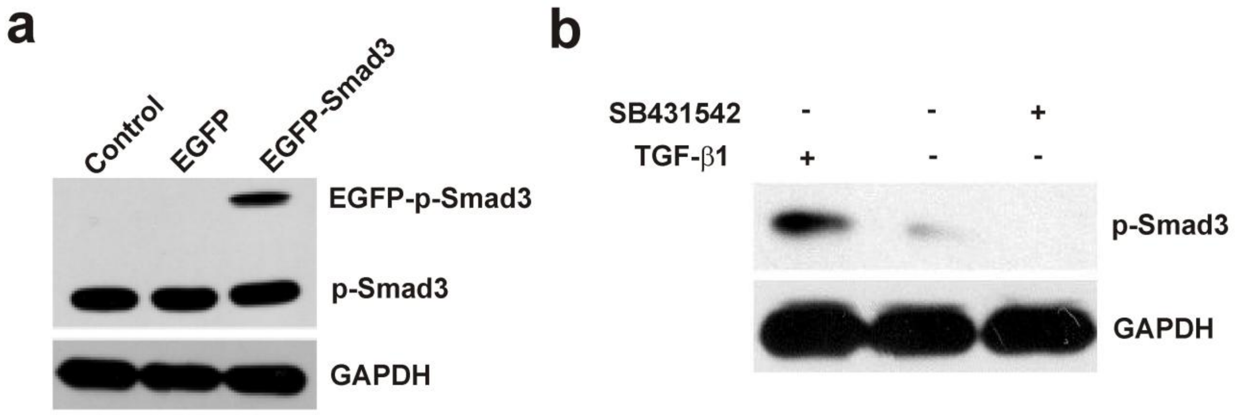

Supplementary Figure 1 Characterization of p-Smad3 level in HeLa cells. (a) Western blot analysis of total cell lysates from non-transfected control cells (Control), EGFP-transfected cells (EGFP) and EGFP-Smad3 transfected cells (EGFP-Smad3). GAPDH was used as the internal control. The result indicated that EGFP-Smad3 can be phosphorylated in response to TGF-β1 as the endogenous Smad3. (b) Western blot analysis of total cell lysates before and after the cells (untransfected) were pre-treated by SB431542 to inhibit the phosphorylation of Smad3. GAPDH was used as the internal control. The result confirmed the specificity of the p-Smad3 antibody used in our study.

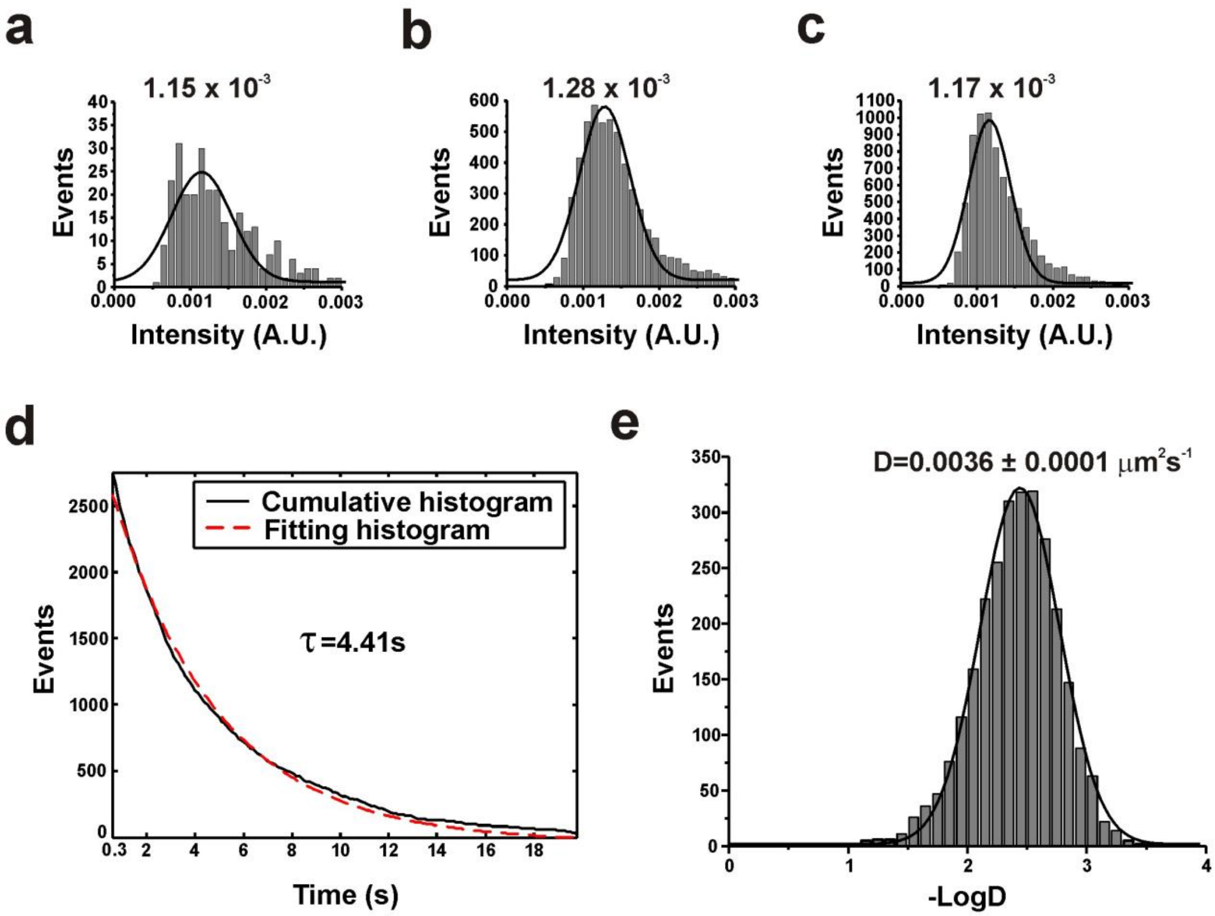

Supplementary Figure 2 Comparison of the intensity of individual membrane docked EGFP-Smad3 molecules to that of single EGFP molecules immobilized on coverslips by TIRFM imaging. (a-c) Fluorescence intensities of diffraction-limited single EGFP molecules fixed on the coverslip (a) and of EGFP-Smad3 molecules docking to cell membrane in HeLa cells in absence (b) and presence of TGF-β1(c). The solid curves showed the fitting of Gaussian function, and the numerical values indicate the peak positions of the fitting curves. (d) Cumulative histograms (solid) of photo-bleaching times of individual EGFP molecules on the coverslip. The dwell time of single EGFP molecules before photo-bleaching was measured. The decay time constant is 4.41 s determined by a single exponential decay fitting function (fitting curve: dotted line). The correlation coefficient of the fitting is above 0.97. (e) Diffusion rates (D) of single EGFP molecules on the coverslip with the marked D value.

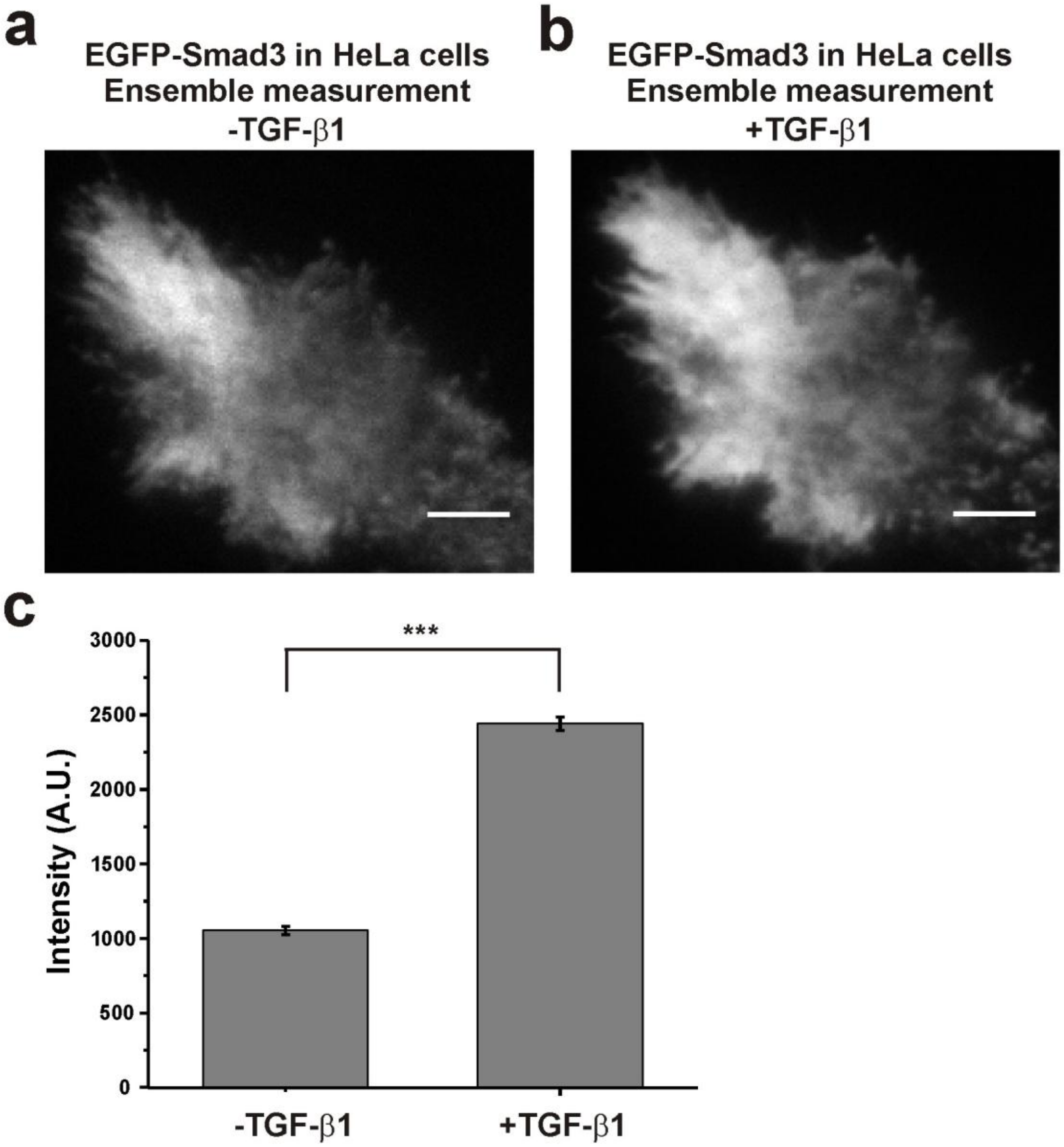

Supplementary Figure 3 TGF-β1 induced the fluorescence enhancement due to the docking of Smad3 to the cell membrane. (a and b) Two typical TIRFM fluorescence images of EGFP-Smad3 on the surface of the living HeLa cells with relatively high EGFP-Smad3 expression, showing fluorescence increase after TGF-β1 stimulation. Scale bar: 5 μm. After the cells were transfected with EGFP-Smad3 for 10 hours, they were imaged by TIRFM before (a) and after (b) TGF-β1 stimulation. (c) The averaged fluorescence intensity of the cell membrane showed in (a) and (b) was quantified and compared. Data are mean intensity ± SEM from 6 cells. (*** $p<0.001$, t-test)

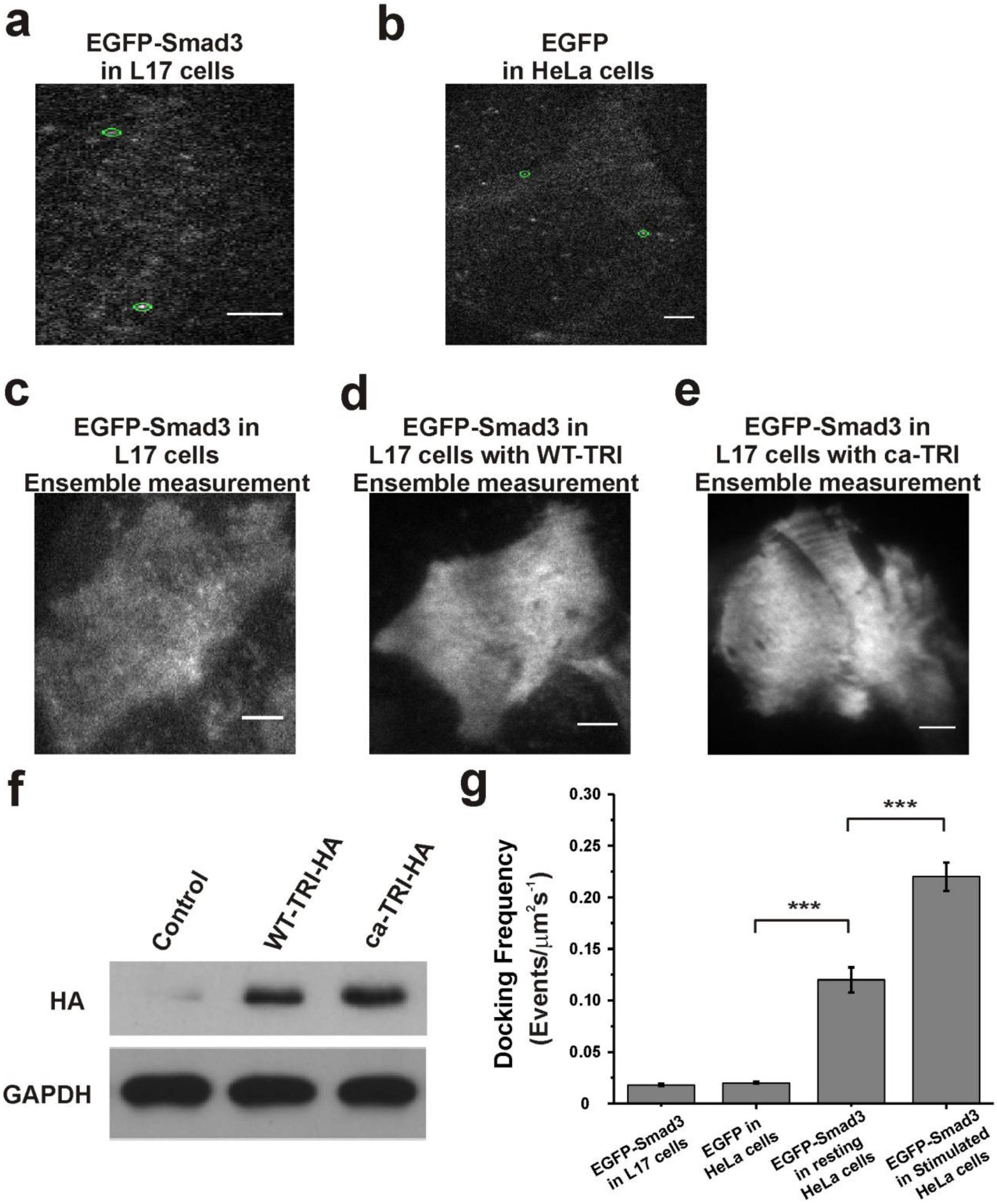

Supplementary Figure 4: Characterization of the membrane-docking dynamics of EGFP-Smad3 molecules in TRI-deficient L17 cells in the absence of TGF-β1 stimulation. (a) A typical single-molecule image of EGFP-Smad3 molecules (enclosed in green circles) on the L17 cell

membrane at the resting state. Scale bar: 5 μm. (b) A typical single-molecule image of EGFP molecules (enclosed in green circles) on the HeLa cell membrane at the resting state. Scale bar: 5 μm. (c-e) A typical image of EGFP-Smad3 at a relatively high expression level (transfected for 10 hours) on the membrane of L17 cells (c), L17cells expressing wide type TGF-β type I receptors (WT-TRI) (d) and L17 cells expressing constitutively activated TGF-β type I receptor (ca-TRI) (e) respectively. Scale bars: 5 μm. (f) Western blot analysis of wt-TRI-HA and ca-TRI-HA using the HA antibody to confirm the expression of TRI in the transfected L17 cells. GAPDH was used as the internal control. (g) Quantification of the docking frequencies of EGFP (0.020 ± 0.001 events/$\mu m^2 s^{-1}$) in HeLa cells and EGFP-Smad3 (0.018 ± 0.001 events/$\mu m^2 s^{-1}$) in L17 cells respectively, and compared the docking frequencies to that of EGFP-Smad3 docking on the plasma membrane of Hela cells before and after TGF-β1 treatment. Data are mean frequency ± SEM from 8 cells (*** $p<0.001$, t-test).

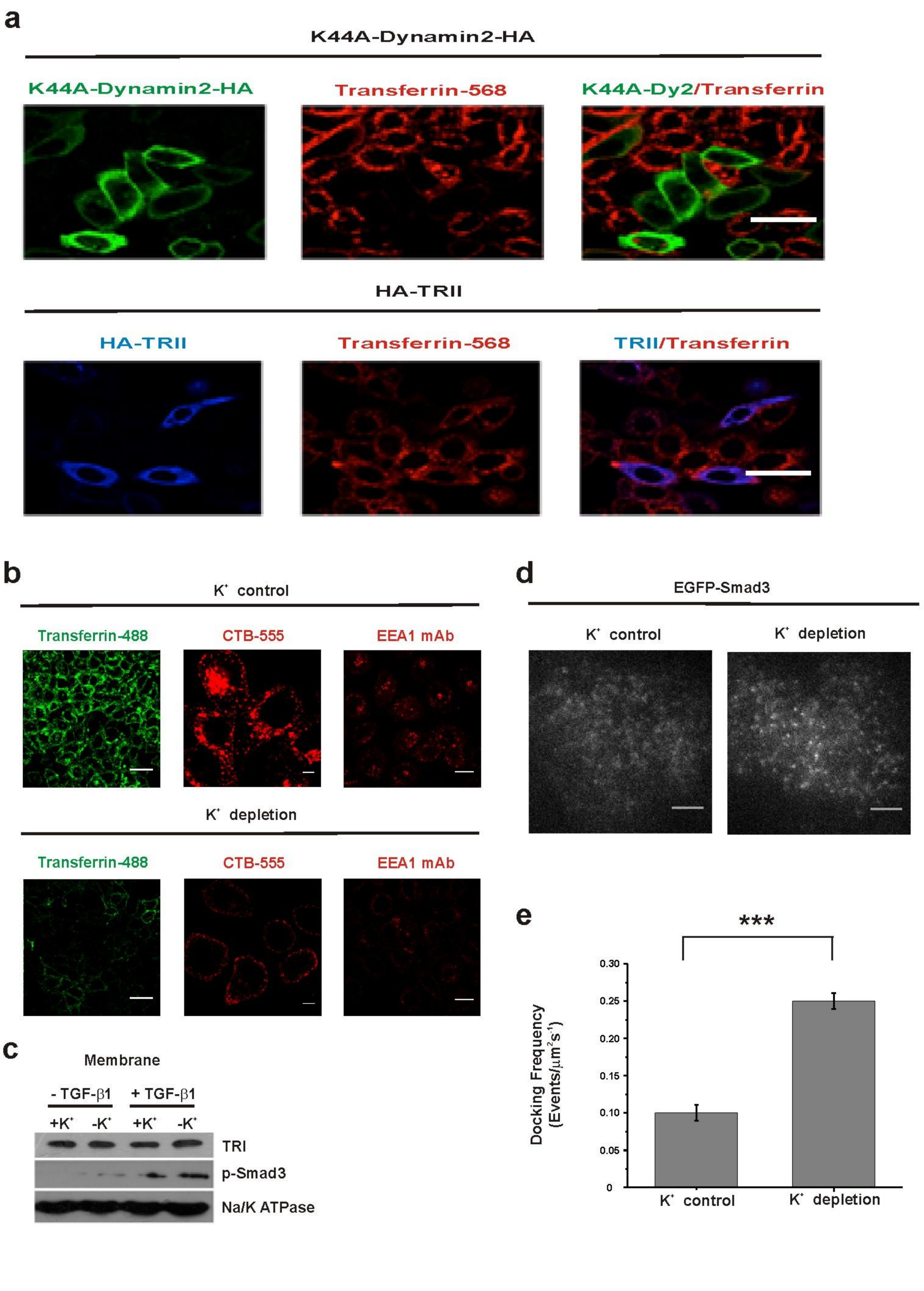
a
K44A-Dynamin2-HA
K44A-Dynamin2-HA
Transferrin-568
K44A-Dy2/Transferrin
HA-TRII
HA-TRII
Transferrin-568
TRII/Transferrin
b
K+ control
Transferrin-488
CTB-555
EEA1 mAb
K+ depletion
Transferrin-488
CTB-555
EEA1 mAb
c
Membrane
- TGF-β1
+ TGF-β1
+K+
-K+
+K+
-K+
TRI
p-Smad3
Na/K ATPase
d
EGFP-Smad3
K+ control
K+ depletion
e
***
Docking Frequency (Events/μm²s⁻¹)
0.30
0.25
0.20
0.15
0.10
0.05
0
K+ control
K+ depletion

Supplementary Figure 5: inhibition of Endocytosis by using K44A-dynamin2-HA and potassium depletion showed that endocytosis is not essential for the membrane docking and activation of Smad3. (a) The effect of K44A-dynamin2-HA on endocytosis was confirmed by confocal microscopy imaging of Alexa 568-conjugated transferrin. HeLa cells expressed with HA-TRII were designed as a control experiment to exclude the effect of transfection on endocytosis. HeLa cells were incubated with transferrin-568 for 15 min, and then washed, fixed, and immunostained with antibody against HA. Confocal images showed that transferrin-568 was internalized into the HeLa cells expressing TRII-HA and for cells expressing K44A-dynamin2-HA, no transferrin-568 was uptaken in cells. Scale bars: 5 μm. (b) The effect of potassium depletion on endocytosis was confirmed by confocal microscopy imaging of Alexa 488-conjugated transferrin, Alexa 555-conjugated CTB and the level of EEA1 (mAb). HeLa cells were pretreated by potassium depletion or the control medium, and then incubated with transferrin-488 and CTB-555 for 15 min or immunostained with the antibody against EEA1. Confocal images showed that the uptake of transferrin-488 and CTB-555 was greatly affected by potassium depletion treatment. And the level of EEA1 was also affected. Scale bars: 5 μm. (c) The levels of p-Smad3 in the membrane extraction were examined by western blotting before and after potassium depletion treatment both in unstimulated and stimulated HeLa cells. Na/K ATPase was used as the internal control. After TGF-β1 stimulation, the level of p-Smad3 was increased in the membrane extraction of the cells with potassium-depletion. (d) TIRFM images of EGFP-Smad3 docking on the plasma membrane of the living HeLa cells before and after potassium depletion treatment. Scale bars: 10 μm. (e) Comparison of the docking frequency of EGFP-Smad3 on the plasma membrane of HeLa cells before and after potassium depletion. The total number of single EGFP-Smad3 molecules docking on cell membrane was counted for 30 s and calculated as docking frequency per μm^2 per s for every single cell. Data are shown as mean frequency ± SEM from 8 cells. The docking frequencies of EGFP-Smad3 were 0.10 ± 0.01 and 0.25 ± 0.01 events/$\mu m^2 s^{-1}$ in the potassium control and potassium depletion medium treated HeLa cells, respectively. (*** $p<0.001$, t-test).

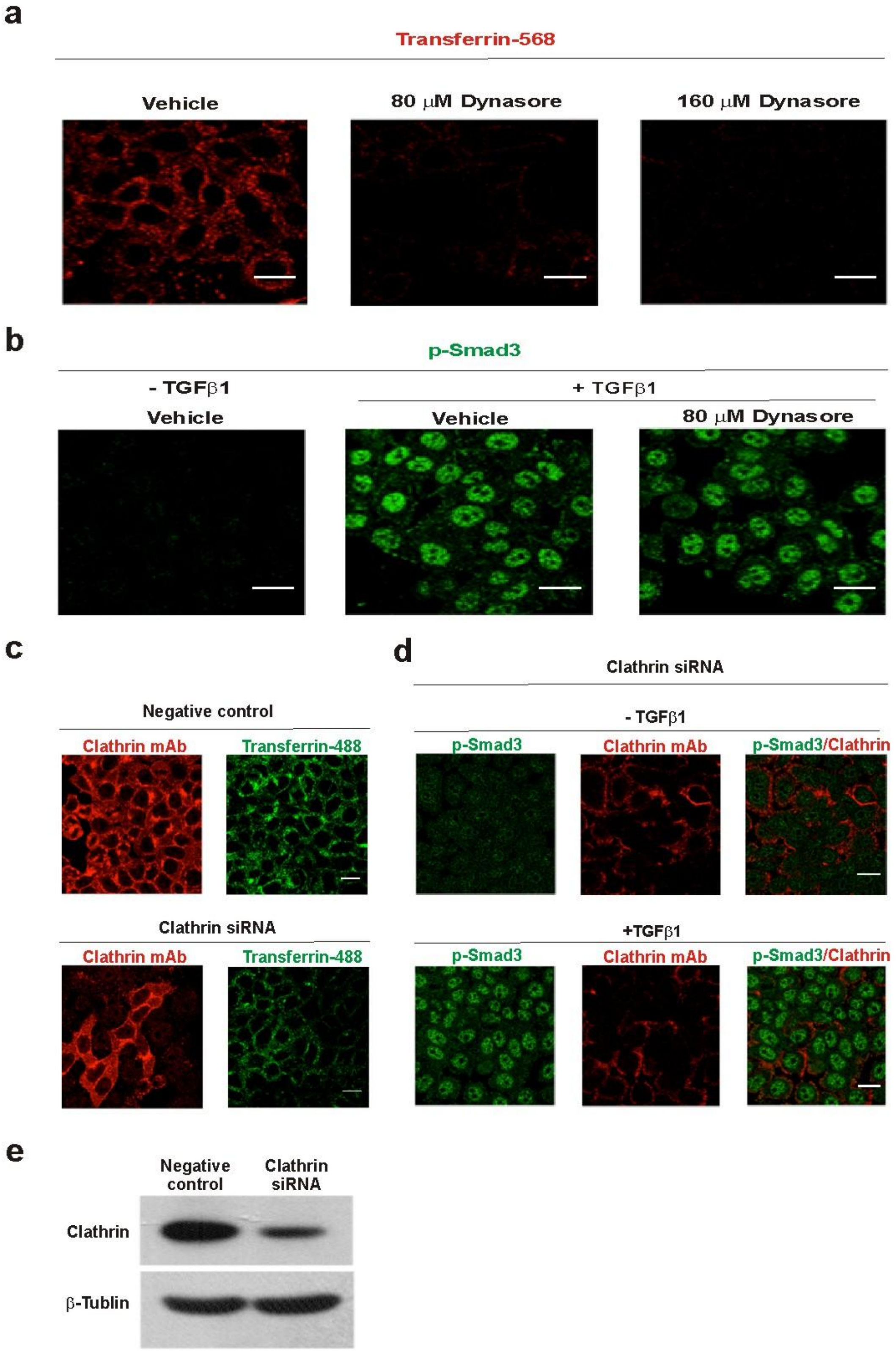
a
Transferrin-568
Vehicle
80 μM Dynasore
160 μM Dynasore
b
p-Smad3
- TGFβ1
Vehicle
+ TGFβ1
Vehicle
80 μM Dynasore
c
Negative control
Clathrin mAb
Transferrin-488
Clathrin siRNA
Clathrin mAb
Transferrin-488
d
Clathrin siRNA
- TGFβ1
p-Smad3
Clathrin mAb
p-Smad3/Clathrin
+TGFβ1
p-Smad3
Clathrin mAb
p-Smad3/Clathrin
e
Negative control
Clathrin siRNA
Clathrin
β-Tublin

Supplementary Figure 6: Inhibition of endocytosis by using dynasore and clathrin siRNA showed that endocytosis is not essential for the membrane docking and activation of Smad3. (a) The effect of Dynasore on endocytosis was confirmed by confocal microscopy imaging of Alexa 568-conjugated transferrin. HeLa cells were incubated with Dynasore at the indicated concentrations (80, 160 μM) for 2 hours, and then incubated with transferrin-568 for 15 min. Confocal images showed that the uptake of transferrin-568 was greatly affected by 80 μM Dynasore. Scale bars: 5 μm. (b) The effect of Dynasore on Smad3 phosphorylation and nuclear transportation was also checked by confocal microscopy. HeLa cells pretreated with Dynasore were immunostained with the antibody against p-Smad3. Confocal imaging of p-Smad3 (pAb, Alexa 488) before and after TGF-β1 stimulation showed that the accumulation of p-Smad3 in nucleus was independent of endocytosis. Scale bars: 5 μm. (c) The effect of clathrin siRNA on endocytosis was confirmed by confocal microscopy imaging of Alexa 488-conjugated transferrin. After transfected with the clathrin siRNA for 24 hours, HeLa cells were incubated with transferrin-488 for 15 min. And then washed, fixed and immunostained with the antibody against clathrin (mAb, Alexa-555). Confocal images showed that the uptake of transferrin-488 was greatly affected by clathrin siRNA. Scale bars: 5 μm. (d) The effect of clathrin siRNA on Smad3 phosphorylation and nuclear transportation was also checked by confocal microscopy. After transfected the clathrin siRNA for 24hours, HeLa cells were immunostained with the antibody against p-Smad3. Confocal imaging of p-Smad3 (pAb, Alexa-488) before and after TGF-β1 stimulation showed that the accumulation of p-Smad3 in nucleus was independent of endocytosis. Scale bars: 5 μm. (e) Western blot analysis of clathrin to confirm the effect of the clathrin siRNA. HeLa cells were treated with clathrin heavy-chain-specific siRNA and the control siRNA, then harvested and subjected for western blotting analyses of clathrin. β-Tublin was used as the internal control.

Supplementary Movies:

Supplementary Movie 1: Imaging of single EGFP-Smad3 molecules on the plasma membrane of live

HeLa cells 5 hours after transfection. Scale bar: 5 μm.

Supplementary Movie 2: Imaging of single EGFP-Smad3 molecules on the plasma membrane from the living HeLa cells 5 hours after transfection followed by TGF-β1 stimulation. Scale bar: 5 μm.

Supplementary Movie 3: Imaging of single EGFP-Smad3 molecules on the plasma membrane from the living HeLa cells 5 hours after transfection followed by SB431542 treatment. Scale bar: 5 μm.

Supplementary Movie 4: Imaging of single EGFP-M-Smad3 molecules on the plasma membrane from the living HeLa cells 5 hours after transfection. Scale bar: 5 μm.

Supplementary Movie 5: Imaging of single EGFP-Smad3 molecules on the plasma membrane from the living HeLa cells 5 hours after transfection. Receptor endocytosis was blocked by transfecting K44A-dynamin2-HA. Scale bar: 5 μm.

References

1 Di Guglielmo, G. M., Le Roy, C., Goodfellow, A. F. & Wrana, J. L. Distinct endocytic pathways regulate TGF-β receptor signalling and turnover. *Nat. Cell Biol.* **5**, 410-421, (2003).

2 Zhao, B. *et al.* PICK1 promotes caveolin-dependent degradation of TGF-β type I receptor. *Cell Res.* **22**, 1467-1478, (2012).
